# Prevalence of impacted teeth and supernumerary teeth by radiographic evaluation in three Latin American countries: A cross-sectional study

**DOI:** 10.4317/jced.57757

**Published:** 2021-04-01

**Authors:** Sindy Tetay-Salgado, Luis-Ernesto Arriola-Guillén, Gustavo-Armando Ruíz-Mora, Aron Aliaga-Del Castillo, Yalil-Augusto Rodríguez-Cárdenas

**Affiliations:** 1 Lecture student o the Division of Oral and Maxillofacial Radiology, School of Dentistry, Universidad Científica del Sur, Lima, Perú; 2 Ph.D. and Associate Professor of the Division of Orthodontics and Division of Oral and Maxillofacial Radiology, School of Dentistry, Universidad Científica del Sur, Lima, Perú; 3 Ph.D. and Associate Professor of the Division of Oral and Maxillofacial Radiology, School of Dentistry, Universidad Científica del Sur, Lima, Perú; 4 Ph.D. Student. Department of Orthodontics. Bauru Dental School. University of São Paulo, Brazil; 5 Ph.D. and Associate Professor of the Division of Oral and Maxillofacial Radiology, School of Dentistry, Universidad Científica del Sur, Lima, Perú

## Abstract

**Background:**

Supernumerary teeth are those that exceed the usual dental formula. There are different classifications depending on their anatomical location, shape and number. The objective of this study was to determine the prevalence of impacted and supernumerary teeth in a sample of panoramic radiographs from three Latin American countries.

**Material and Methods:**

A retrospective and cross-sectional study with a quantitative approach, in which the radiographic observation and analysis of 2000 panoramic radiographs, provided by different radiological centers in Peru, Colombia and Bolivia were performed. An examiner, specialized in Stomatology and Oral Surgery, carried out the radiographic analysis to detect the presence of impacted teeth and supernumeraries teeth. All the images were evaluated in a dark room in digital format. The SPSS version 23 package was used for statistical analysis. The Chi-square test was used to determine the association of age, sex, and nationality with the presence of impacted or supernumerary teeth. The level of significance was *p*< 0.05.

**Results:**

The prevalence of impacted teeth was 1.7%, with the upper left canine being the most frequent (58.85%). The prevalence of supernumeraries was 3.15% (76 cases), the most frequent being the mesiodens with 1.7% (34), and Peru showing the highest prevalence of mesiodens. No association was found regarding sex.

**Conclusions:**

The prevalence of impacted teeth evaluated in three recognized radiologic centers from three Latin American countries was low, with the upper left canine being the most frequently impacted tooth. Likewise, the prevalence for supernumerary teeth was also low, with mesiodens having the highest prevalence.

** Key words:**Supernumerary tooth, impacted tooth, panoramic radiograph.

## Introduction

Dental anomalies are congenital malformations of the dental tissues that are presented by alterations in development, which may include the shape, number, size, position, eruption, space and structure (1). Impacted teeth (IT) are those which, after having reached the normal period of eruption, are trapped inside the maxillaries maintaining the integrity of their physiological peri-coronary sac. The prevalence of these teeth is high in young and adult populations. The most common local causes include the density of the bone that covers the tooth, lack of space in underdeveloped maxillaries, prolonged retention of temporary teeth or their premature loss, irregularity in the pressure and position of an adjacent tooth. However, the most common cause is gingival fibrosis (1-3). An impacted tooth is completely or partially covered by mucosa and bone for more than 2 years after the time of physiological eruption. Teeth impaction is a commonly observed dental anomaly, although the prevalence varies from place to place and from tooth to tooth. Third molars are the teeth most frequently impacted followed by the maxillary canines.

The etiology of IT is varied and multifactorial, including local or systemic factors. Local factors associated with IT are lack of space in the dental arch, root dilaceration, trauma, ankylosis of the primary teeth, mesialization of the teeth as a result of premature loss of the primary teeth, ectopic positioning of the teeth buds, and inflammatory or pathological lesions, among others. Systemic factors associated with IT are incorrect nutrition, anemia, rickets, vitamin D deficiency, endocrine diseases, syndromes, and specific infections such as syphilis and tuberculosis, among others. Genetic factors may also play a role in these conditions (2-4).

Supernumerary teeth (ST) or hyperodontia is an abnormality of the number and shape in which there is an increase in the normal number of dental organs. This pathology more frequently occurs in men than in women with a ratio of 2:1, and a higher number of cases are reported in the maxilla than in the mandible with a ratio of 8:1. In addition, ST more frequently occur in the upper midline and in relation to the environment, approximately 5% more ST are impacted (4).

The etiology of ST is unknown; however, some theories have attempted to explain their development. The most accepted theory is hyperactivity of the epithelial cells of the dental lamina and the theory of excision or dichotomy of the dental follicle. In relation to the first theory, it is believed that during the evolution of the dental germ, degeneration of the dental lamina occurs, leaving remnants of this in the maxillaries, generating the subsequent development of hyperodontia, while in the second theory, the follicle is divided into two parts, one corresponding to the normal tooth and the other to the ST (5).

Likewise, the presence of ST may be associated with genetic syndromes such as cleidocranial dysplasia, Ehlers Danlos, Down, Fabry Anderson or the Gardner syndrome (5). ST are classified in different ways according to the position. They can be mesiodens (when located between the central incisors), lateral incisors (when located in the lateral incisor area), premolars (located in the premolar area), paramolars (located in the molar area) and distomolars (located distal to the third molar) (6). According to morphological characteristics they are divided into supplementary, that is, extra teeth that have normal characteristics in shape and size to the adjacent teeth, and rudimentary teeth, which present abnormalities in shape and size to the adjacent teeth. The latter can be conical, tubercular or molariform according to the environment and are classified as included and erupted (7,8). Pathologies such as odontoma, cementoblastoma and osteoma must be taken into account as differential diagnoses (9). The prevalence of ST in the general population varies from 1% to 3%. Mesiodens occur in 45% to 47% of ST cases, while premolar ST represent 8% to 10%, and approximately 40% of ST are paramolar. Mesiodens were the most frequent type of ST, with the maxilla being the most affected (10-12).

In Latin America, an epidemiological profile of IT and ST, which allows evaluating the scope and frequency of these events in the Latino population, has not yet been established. This information is necessary to determine the possible variations in the frequencies of these alterations among different Latin American countries. Therefore, the aim of this study was to determine the prevalence of IT and ST in three Latin American countries (Colombia, Peru and Bolivia) with the use of radiographic studies performed in three recognized diagnostic centers.

Material and Methods

This retrospective and cross-sectional observational study was approved by the Institutional Commission of Ethics and Research of the School of Stomatology of the Científica del Sur University (Peru). Since this study aimed to evaluate panoramic radiographs acquired in diagnostic imaging centers of subjects attended for reasons unrelated to the study, no ethical aspects were involved Likewise, confidentiality of the personal data of the patients studied was maintained, with the names, surnames, and the data of any radiography that could lead to the identification of any of the subjects remain unknown, ensuring strict confidentiality of the study sample.

-Sample size

The population consisted of radiographs of patients who were attended in three private radiographic centers (the Centro de Diagnostico por Imagenes CDI in Lima, Peru; Centro Radiografico Cerpax in Cochabamba, Bolivia and Centro Radiografico Orlando Martínez in Cartagena, Colombia) to undergo panoramic radiographs during 2017-2018. Before starting the study, 750 panoramic radiographs (250 per center) were randomly requested from the participating centers to carry out a pilot test. With the results of the pilot test it was determined the sample size. A total of 1085 panoramic radiographs were required to estimate a prevalence of mesiodens of 5%, since these were the ST most frequently found with a confidence level of 95% and an error of 5%. However, a total of 2,000 radiographs were finally obtained (667 from Peru, 530 from Colombia and 803 from Bolivia).

-Selection criteria

The sample included randomly selected radiographs of patients between 12 and 55 years of age from the three participating diagnostic centers. Radiographs showing orthodontic treatment, third molars, total absence of dental organs, oral rehabilitation in the anterior sector, maxillofacial trauma, syndromes involving bone disorders and patients with maxillofacial tumors were excluded from the study.

-Processing and analysis technique

The panoramic radiographs were obtained with Planmeca equipment and were analyzed in digital format. The radiographic analysis was performed by quadrant, starting with the upper right quadrant and ending with the lower right quadrant, evaluating the presence of IT, ST and mesiodens teeth. The age and sex of the individual and the origin of each radiograph were noted.

-Measurement of variables

Likewise, the main researcher was trained with two expert radiologists (GRAM and YARC) and two orthodontists (LEAG and AADC) in the diagnosis of ST and IT. We then determined the intra-observer reliability of the main researcher, a dentist specialist in Stomatology and Oral Surgery with skills in radiographic interpretation, obtaining a Kappa greater than 0.80 in both impaction diagnoses.

In this study socio-demographic variables were evaluated including age, sex, and origin. Dental anomalies were defined as the presence or absence of IT, ST, type, and the quantity and location of these teeth were evaluated using the classification of Santosh and Sneha. (6)

-Statistical analysis

The study data were tabulated in a matrix Table of the statistical program IBM SPSS statistics (version 24.0; IBM, Armonk, NY). Descriptive analyses were performed using frequencies and percentages assuming 95% confidence intervals. The Chi-square test was used to determine relationships among variables, and a *p* value < 0.05 was considered statistically significant.

## Results

A total of 3140 panoramic radiographs were evaluated with 1140 being discarded for not meeting the inclusion criteria. Finally, a total of 2000 radiographs were included in the study. Of these, 51.3% corresponded to female sex and 48.7% were from males. Regarding the country of origin, 530 radiographs were from Colombia, 667 from Peru and 803 from Bolivia. According to age, the highest frequency ranged from 11 to 20 years with 38.5%.

-Prevalence of impacted and supernumerary teeth

The prevalence of IT was 1.3% (34 teeth in 26 patients), the most frequently involving the upper left canine (58.85%). Table 1 shows the presence of mesiodens in the radiographs from the three radiological centers, with 34 (2.8%) of the 2000 radiographs evaluated demonstrating mesiodens, which were most frequently found in radiographs from Peru (*p*=0.019). Furthermore, Table 1 shows the presence of ST in the radiographs evaluated, with 29 (1.5%) of the 2000 radiographs showing ST, with a similar prevalence of ST being observed among the three study centers (*p*=0.599).

**Table 1:**
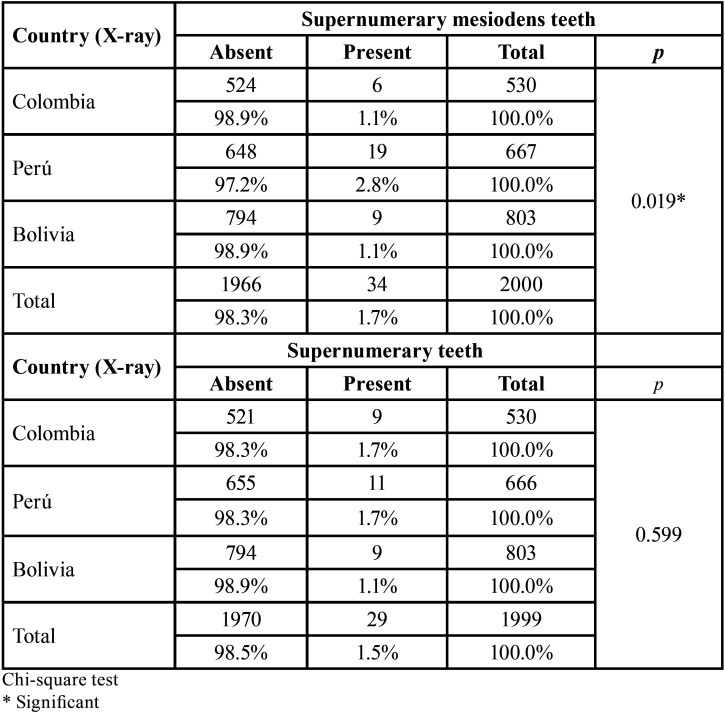
The presence of supernumerary and mesiodens teeth in the panoramic radiographs evaluated from radiological centers in three Latin American countries.

Table 2 describes the eruption status of the mesiodens ST in the panoramic radiographs evaluated from the three radiological centers. This tooth was found in 63.2% to 83.3% of the radiographs evaluated, with no differences according to the origin of the radiograph.

**Table 2:**
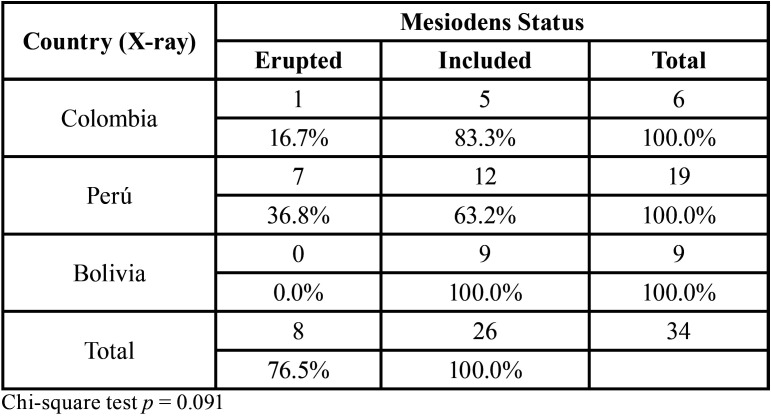
Eruption status of the supernumerary mesiodens teeth in the panoramic radiographs evaluated from radiological centers in three Latin American countries.

Furthermore, in the radiographs evaluated, it was found that the most frequent number of ST per case was one tooth, with no differences in the country of origin (Table 3). Finally, the most frequent ST types found in this sample were paramolars and premolars, although distomolar ST were found in 33.3% of the radiographs from Bolivia (Table 4).

**Table 3:**
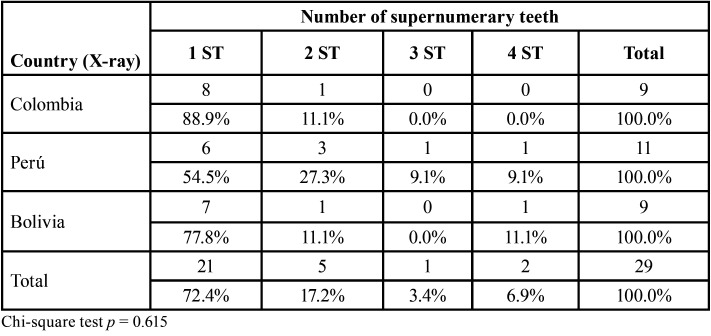
Number of supernumerary teeth in the panoramic radiographs evaluated from radiological centers in three Latin American countries.

**Table 4:**
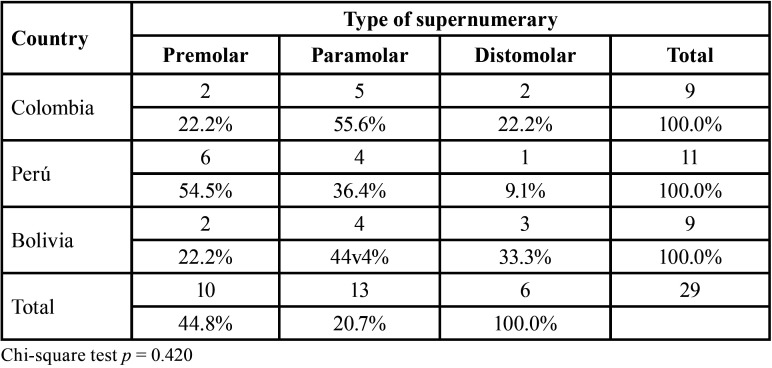
Evaluation of supernumerary mesiodens tooth type in panoramic radiographs from radiological centers in three Latin American countries.

## Discussion

IT and ST are a frequent eruption anomaly that dentists must be trained to diagnose and provide correct treatment. Radiographic studies and clinical examination must be performed to diagnose these anomalies. Besides, clearly the second most frequent IT were maxillary canine teeth which coincides with the results of the present study (6,7,13). Also, these teeth are by far one of the most frequent causes of IT in orthodontics. On the other hand, the prevalence of ST can vary according to the geographical setting, which in the case of Latin America is very varied. In this regard, Lara *et al.* (12) concluded that there was a low prevalence of mesiodens (1.5%) in deciduous and mixed dentition and the condition was not associated with other dental anomalies, except for the maxillary lateral incisor agenesis. In our study, the most frequent ST was mesiodens with 1.7%.

Various studies have found different prevalences regarding our study. In this way, in Europe, Schmuckli *et al.* (14) described ST based on radiographic analysis in a study carried out in Switzerland. They studied 3004 patients, 44 of whom had ST, with a higher prevalence of 1.1% in men and 0.4% in women. In addition, mesiodens were the most frequent ST with 33 cases. Goutham *et al.* (15) evaluated dental anomalies in a population in India, they analyzed 1080 panoramic radiographs, finding a prevalence of ST of 35.27%. Moreover, Dang *et al.* (16) studied dental abnormalities using panoramic radiographs in a pediatric population in Australia. They prospectively reviewed 1050 radiographs and reported that 5.14% of the patients had a dental anomaly, with 0.28% presenting ST. Laganà *et al.* (17) in Rome in a sample of 4076 panoramic radiographs concluded the prevalence of mesiodens was 0.66%, which is not far from the 1.7% that found in the present study. Besides, Chen *et al.* (18) found that mesiodens were more common ST. They found 185 ST in the maxilla and 56 in the mandible, 153 were mesiodens and 115 were inverted. However, Brinkmann *et al.* (19) concluded that although mesiodens were considered the most common ST, the present study found distomolars and supernumerary premolars to be the most frequently occurring.

In our study, evaluating panoramic radiographs from three Latin American countries (Peru, Colombia and Bolivia) we found some differences with respect to the aforementioned studies, although the prevalence of ST remained low, involving 3.8% of the 2000 cases studied. These variations in prevalence could be explained by great differences in sample size from study to study. Regarding the increase in ST according to sex, our study coincides with the frequency of appearance of the aforementioned studies, with male sex being the most affected. In this way, the importance of this study was established an epidemiological profile of IT and ST by evaluating panoramic radiographs of patients residing in three geographically close Latin American countries. Although the results showed that the prevalence varied between the Peruvian, Bolivian, and Colombian populations, these results could be explained by racial diversity. In the Peruvian and Bolivian populations most people of the descendants are indigenous, respectively, while in Colombia the population has a greater proportion of black population. Besides, further studies are needed to corroborate the results within other cities in the same countries to determine whether the trend remains or shows variations from the low prevalence found in the present study. According to the results of the present study and analysis of the bibliography available, it is highlighted that mesiodens were the most prevalent dental anomaly.

The findings of the present study can promote the adoption of public health policies to avoid impaction of permanent teeth in growing patients with suspicious imaging studies. This is especially important in relation to the upper left canine teeth, due to the high costs related to viability. The extraction of supernumerary teeth in the anterior area can avoid bad eruption or impaction of the permanent canines and decrease the complexity of orthodontic treatment.

-Limitations 

Panoramic radiography is a radiographic dental technique able to achieve well-defined, isomorphic, isometric and orthogonal continuous recordings of all neighboring and complementary teeth and structures, this radiographic technique is essential when it is not possible to obtain intraoral radiographs. In the case of excessive trismus, local inflammatory processes or pronounced nausea, extraoral radiographs must be used. The images obtained in panoramic radiography show a certain degree of magnification. Nonetheless, these images are very useful for diagnostic purposes in dentistry today and especially for the diagnosis of IT and ST.

## Conclusions

The prevalence of IT in the present study was 1.3%, with the most frequent IT being upper left canine, excluding third molars. On the other hand, the prevalence of ST was 3.15%, with mesiodens being the most frequent ST. These prevalences for IT and ST in the three Latin American countries evaluated are low. It should be highlighted that the use of panoramic radiography was an important and easily accessible diagnostic tool for the identification and evaluation of IT and ST.
